# A novel human ex vivo skin model to study early local responses to burn injuries

**DOI:** 10.1038/s41598-020-79683-3

**Published:** 2021-01-11

**Authors:** Elisabeth Hofmann, Julia Fink, Anita Eberl, Eva-Maria Prugger, Dagmar Kolb, Hanna Luze, Simon Schwingenschuh, Thomas Birngruber, Christoph Magnes, Selma I. Mautner, Lars-Peter Kamolz, Petra Kotzbeck

**Affiliations:** 1 COREMED-Cooperative Centre for Regenerative Medicine, JOANNEUM RESEARCH Forschungsgesellschaft mbH, Graz, Austria; 2 Division of Plastic, Aesthetic and Reconstructive Surgery, Department of Surgery, Medical University of Graz, Graz, Austria; 3 HEALTH-Institute for Biomedicine and Health Sciences, JOANNEUM RESEARCH Forschungsgesellschaft mbH, Graz, Austria; 4 Core Facility Ultrastructure Analysis, Center for Medical Research, Medical University of Graz, Graz, Austria; 5 Division of Cell Biology, Histology and Embryology, Gottfried Schatz Research Center, Medical University of Graz, Graz, Austria; 6 Division of Endocrinology and Diabetology, Medical University of Graz, Graz, Austria

**Keywords:** Cell biology, Physiology, Medical research, Molecular medicine

## Abstract

Burn injuries initiate numerous processes such as heat shock response, inflammation and tissue regeneration. Reliable burn models are needed to elucidate the exact sequence of local events to be able to better predict when local inflammation triggers systemic inflammatory processes. In contrast to other ex vivo skin culture approaches, we used fresh abdominal skin explants to introduce contact burn injuries. Histological and ultrastructural analyses confirmed a partial-thickness burn pathology. Gene expression patterns and cytokine production profiles of key mediators of the local inflammation, heat shock response, and tissue regeneration were analyzed for 24 h after burn injury. We found significantly increased expression of factors involved in tissue regeneration and inflammation soon after burn injury. To investigate purely inflammation-mediated reactions we injected lipopolysaccharide into the dermis. In comparison to burn injury, lipopolysaccharide injection initiated an inflammatory response while expression patterns of heat shock and tissue regeneration genes were unaffected for the duration of the experiment. This novel ex vivo human skin model is suitable to study the local, early responses to skin injuries such as burns while maintaining an intact overall tissue structure and it gives valuable insights into local mechanisms at the very beginning of the wound healing process after burn injuries.

## Introduction

Burn injuries are characterized by tissue denaturation and strong local inflammatory responses, which can spread even beyond the burn site. Local inflammatory processes after burn injuries can trigger a systemic inflammatory response manifesting itself as sepsis or as systemic inflammatory response syndrome (SIRS), both being potentially life threatening and causing premature death^1–4^. The local and systemic inflammatory processes after burn injuries are highly complex, and the major aim in burn research is to elucidate the responsible key mediators^5^. In response to burn injuries, numerous factors are locally as well as systemically increased including heat shock proteins, e.g. HSPA4 (former HSP70)^6^ or SERPINH1 (former HSP47)^7^, the transcription factor HIF1A (hypoxia inducible factor 1 subunit alpha)^8^, as well as KRT17 (keratin 17)^9,10^. Numerous pro- and anti-inflammatory mediators such as the interleukins IL1B, IL6^11,12^, C-X-C motif chemokine ligand 8 (CXCL8; former IL8)^13^, and IL1 receptor antagonist (IL1RN; former IL1RA)^14^, are produced in a strictly regulated but highly dynamic manner. Moreover, factors involved in angiogenesis, such as vascular endothelial growth factor alpha (VEGFA)^15^, CXCL12 (former stromal-derived factor 1, SDF-1)^16^, fibroblast growth factor 2 (FGF2, former bFGF)^17,18^ or leptin (LEP)^19–22^ are deregulated upon burn injury. Additional growth factors including transforming growth factor beta 1 (TGFB1)^23^ and the cell motility protein actin alpha 2, smooth muscle (ACTA2; alpha smooth muscle actin, aSMA)^24^ are known to be up-regulated in the course of burn wound healing.

However, most studies investigating the factors involved in burn injury mediated inflammation and wound healing pathways rely on the analyses of serum samples and thus explore systemic but not local changes^25–30^. Reliable burn wound models are needed in order to study the exact sequence of local inflammatory responses and healing processes and how they trigger burn injury complications. Several rodent and porcine in vivo models for burn injuries have been described^31^, but due to anatomical and physiological differences, it is difficult to directly translate findings from animal models to the clinical situation^32^. Ex vivo skin organ culture models based on the cultivation of small skin biopsies allow for the analysis of skin pathologies including burn injuries in a fully human situation^32–41^, but they are dependent on rather artificial culture conditions.

The aim of this study was to develop and evaluate a human ex vivo model, which is independent of culture conditions and in which the overall tissue structure is fully maintained. The ex vivo model we describe here does not rely on any culturing methods, but is based on the use of fresh skin explants that are derived from abdominoplasty or circumferential body lift surgeries and that include the subcutaneous tissue. Burn injuries are induced on the fresh explants^42^ and local processes can be monitored for up to 36 h after resection.

We characterized this ex vivo model of burn injuries on the histological as well as on the molecular level. Histological and ultrastructural analyses were used to confirm partial-thickness burn pathology. Heat shock, inflammatory and regenerative responses to burn injury were monitored over a period of 24 h while leaving the normal skin architecture intact, resulting in local time-dependent gene expression and cytokine production profiles. In addition, we analyzed the local responses to a different kind of inflammatory stimulus independent of tissue disruption. Lipopolysaccharide (LPS), a cell wall component of gram-negative bacteria, activates the innate immune system via toll-like receptor signaling^43^. LPS was injected into the dermis of the explants thus mimicking a bacterial infection in the skin. Gene expression analyses were performed to elucidate the effect of the intradermal application of LPS in comparison to the injection of a sterile NaCl solution that served as a control for the injection trauma.

## Results

### The use of fresh skin explants allows for the analysis of local responses to burn injury and inflammatory stimuli

**Figure 1:**
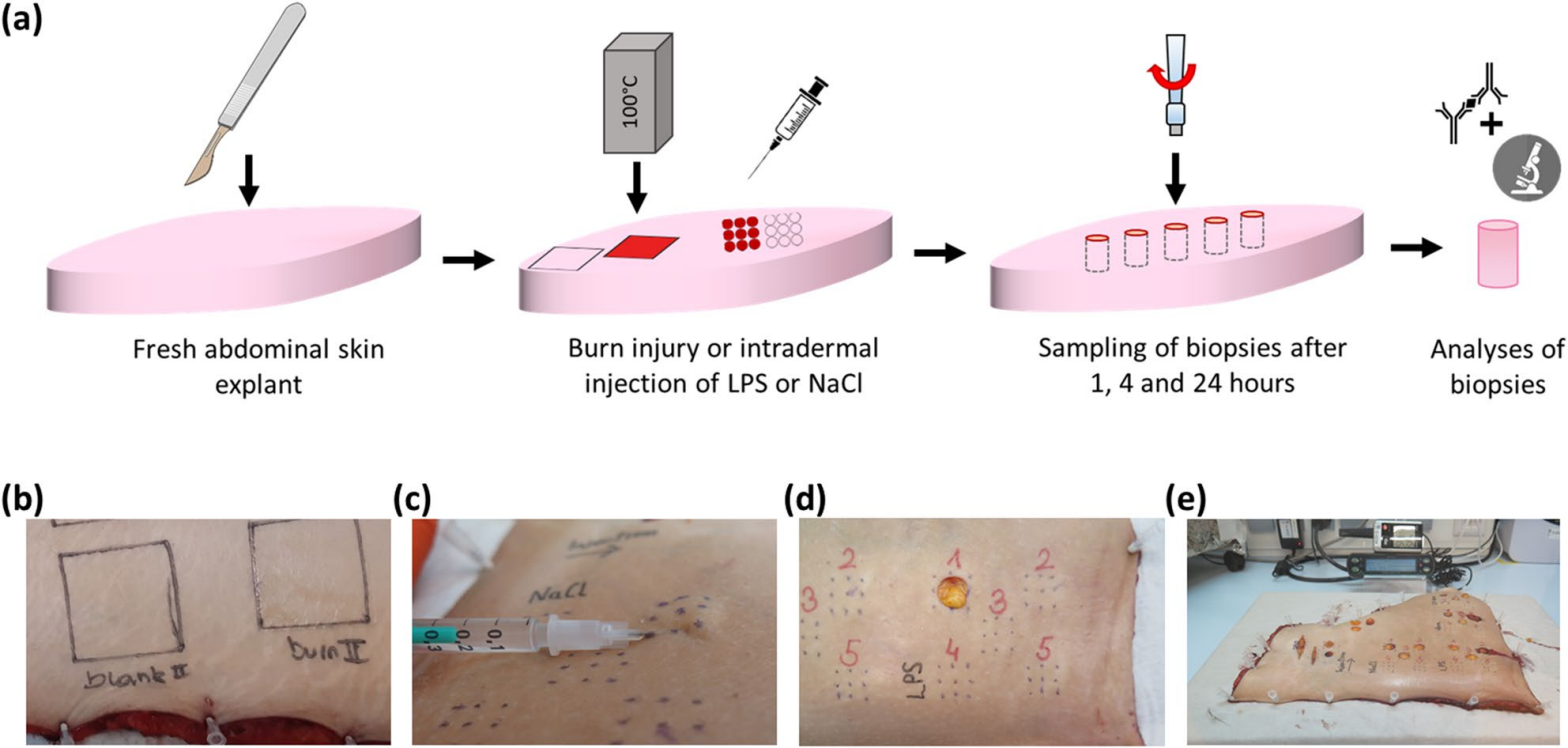
Experimental setup for the ex vivo skin model for burn injury and inflammation. (**a**) The experimental procedure included the immediate transfer of the fresh abdominal skin explants to the laboratory, creation of a burn injury or intradermal injection of LPS or NaCl, collecting punch biopsies and molecular and histological analyses. Representative pictures of (**b**) a burn injury (right field) compared with a healthy, blank site (left), (**c**) the intradermal injection of LPS (inflammation; red circles in (a)) or NaCl (control for injection trauma; open circles in (a)), (**d**) sampling of biopsies and (**e**) the skin explant in the climate chamber.

Burn injuries were induced on fresh human skin explants (Fig. 1). In order to analyze inflammatory reactions independently of tissue denaturation, LPS was injected into the dermis. Blank, unburnt skin sites and NaCl injected sites served as control sites for burn injury and LPS stimulation, respectively. Biopsies were collected at 1, 4, and 24 h after the burn or inflammatory stimulus allowing for the analysis of gene expression patterns and protein production of heat-shock, regenerative and inflammatory key factors as well as of histology and ultrastructure.

### Partial-thickness burns result in significant changes in gene expression of markers for tissue regeneration

**Figure 2:**
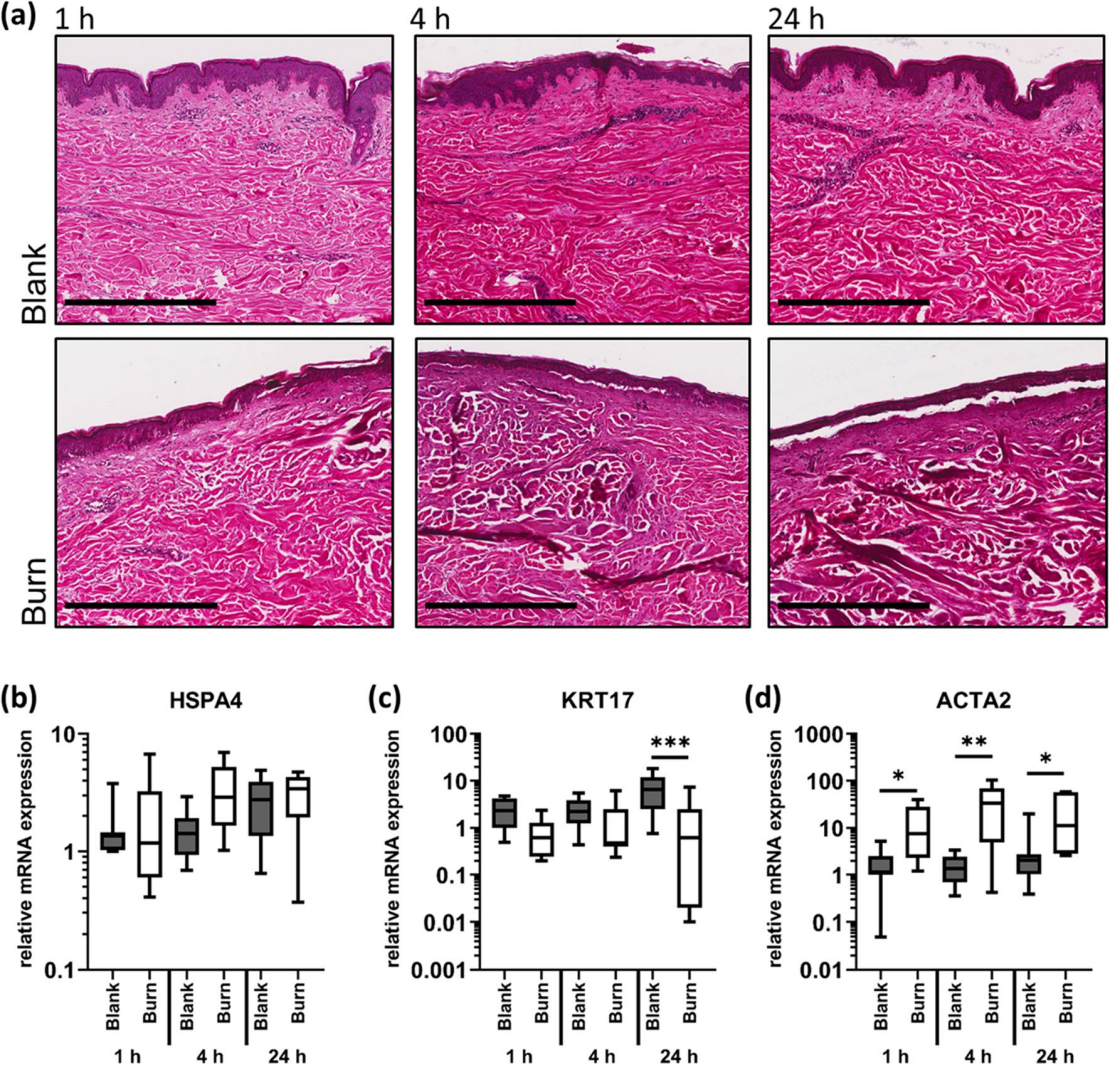
Partial thickness burns affect the expression of HSPA4, KRT17 and ACTA2. Contact burn injuries were created on fresh human skin explants by applying a heated metal block (100 °C). Biopsies were collected after 1, 4 and 24 h. (**a**) Histology confirmed a partial thickness burn pathology. Scale bar = 500 µm. Relative mRNA expression levels of (**b**) HSPA4, (**c**) KRT17, and (**d**) ACTA2 were determined by RT-qPCR, normalizing target gene expression to the averaged expression of RPLP0 and TBP. Data are presented as median (line) the interquartile range (box) and the minimum and maximum values (whiskers) from three individual skin explants. P-values < 0.05 were considered as statistically significant with *, **, *** indicating p < 0.05, p < 0.01, and p < 0.001, respectively (dependent on normal distribution ANOVA or Kruskal–Wallis test).

Histology showed that the burn-induced tissue damage corresponded to a partial-thickness burn pathology (Fig. 2a), with complete disintegration of the epidermis, and partial denaturation of the dermis in comparison to unburnt (blank) skin. In response to the burn injury, gene expression levels of HSPA4 (Fig. 2b) and SERPINH1 (data not shown) were not significantly changed, but HSPA4 showed a trend to be transiently increased after 1 and 4 h. A significant decrease of gene expression levels of KRT17 was observed 24 h after the burn injury (Fig. 2c). Expression levels of TGFB1 were unaffected (data not shown), but those of ACTA2 were significantly increased as soon as 1 h after burn injury (Fig. 2d).

**Figure 3:**
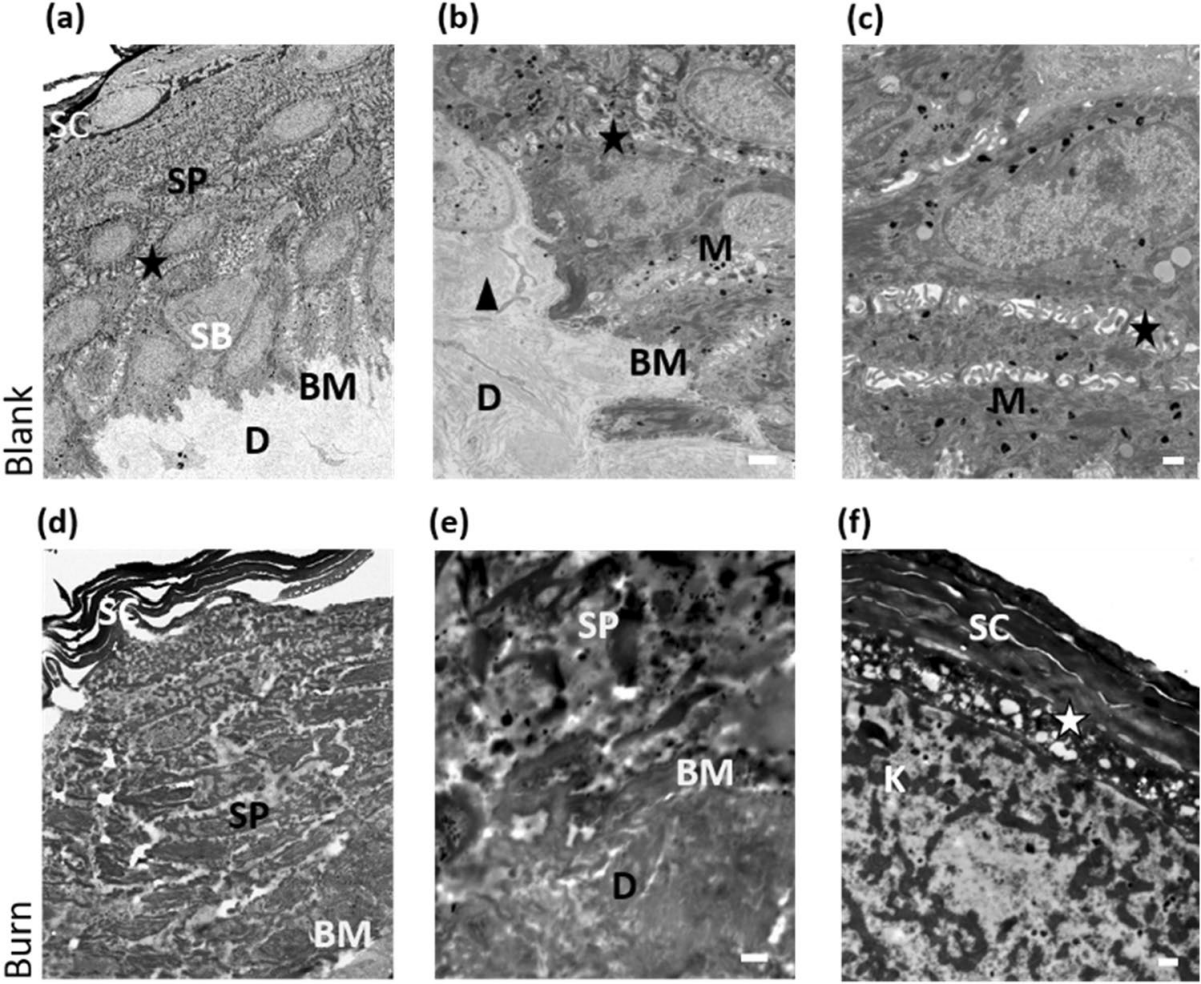
Partial thickness burns disrupt the ultrastructural organization of epidermis and dermis. (**a**) Overview of blank, untreated skin including 25 stitched micrographs (magnification ×1700); SC (*stratum corneum*), SP (*stratum spinosum*) with keratinocytes—(**a**), (**b**), (**c**) asterisks indicate connections of keratinocytes, SB (*stratum basale*) with BM (basement membrane). (**b**), (**c**) detailed TEM micrographs of epidermal–dermal transitions. Areas are rich in M (melanocytes) associated with keratinocytes and are located in the lowest epidermal layer. (**b**) Dermis—arrowhead indicates collagen fibres in various directions. (**d**) Overview of a burnt skin including 25 stitched micrographs (magnification 1700x) showing SC and SP. (**e**), (**f**) detailed TEM micrographs of epidermis of burnt skin. (**e**) BM between SP and dermis is hardly detectable; the borders of all cellular structures are missing. (**f**) *Stratum granulosum* (white asterisk) illustrates blowy inclusions and loose structures. Scale bars: (**b**) 500 nm, (**c**) 1000 nm, (**e**) 500 nm and (**f**) 1000 nm.

Ultrastructural analyses showed that the *stratum corneum* detached from the other epidermal layers upon burn injury. Epidermal structures were condensed (Fig. 3d–f) compared with blank skin (Fig. 3a–c). While the basement membrane separates the *stratum basale* from the dermis in healthy skin, such structures were hardly detectable in burnt skin areas.

**Figure 4:**
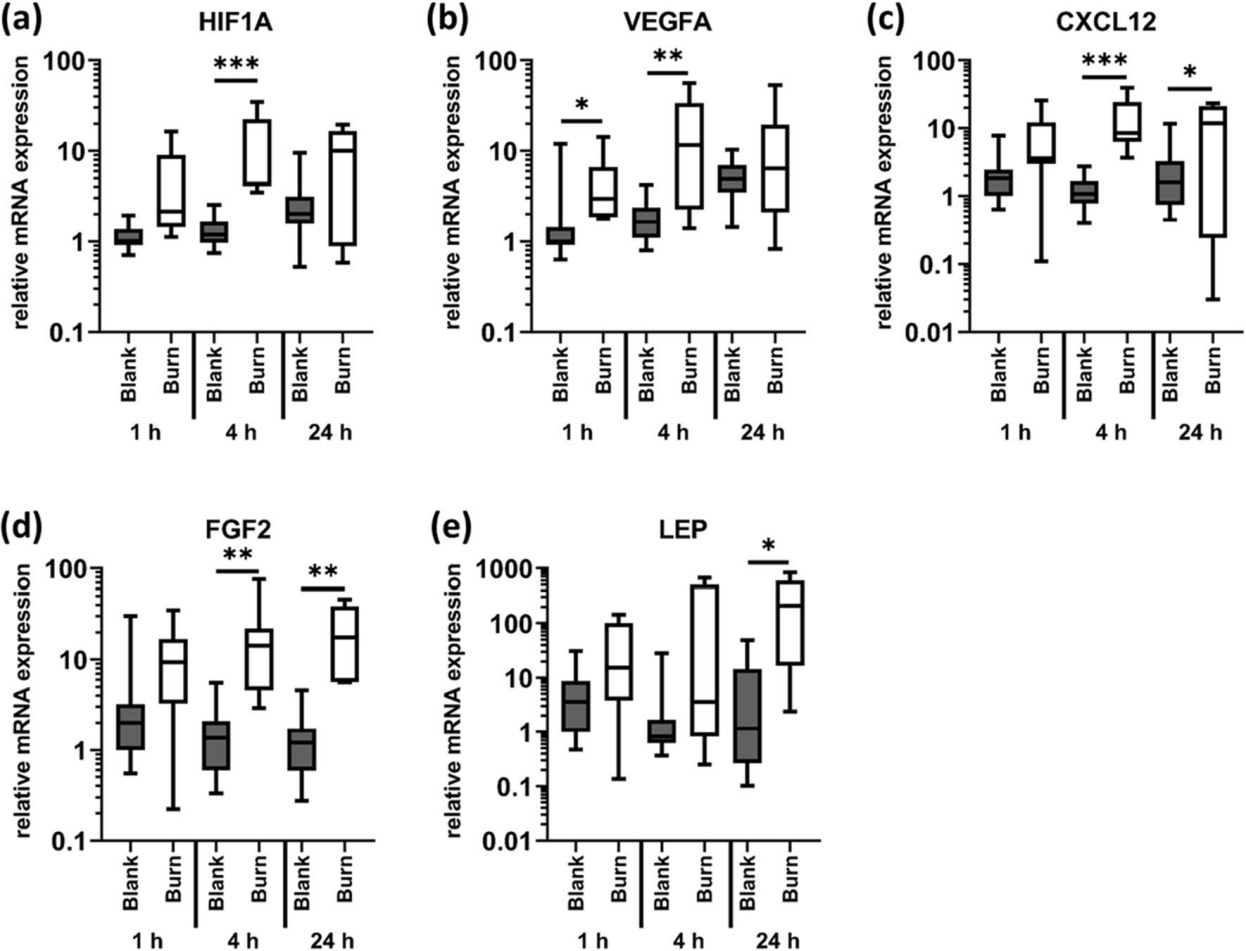
Burn injury strongly enhances the expression of genes involved in angiogenesis. Contact burn injuries were created on fresh human skin explants by applying a heated metal block (100 °C). Biopsies were collected after 1, 4 and 24 h. Relative mRNA expression levels of (**a**) HIF1A, (**b**) VEGFA, (**c**) CXCL12, (**d**) FGF2 and (**e**) LEP were determined by RT-qPCR, normalizing target gene expression to the averaged expression of RPLP0 and TBP. Data are presented as median (line) the interquartile range (box) and the minimum and maximum values (whiskers) from three individual skin explants. p-values < 0.05 were considered as statistically significant with *, **, *** indicating p < 0.05, p < 0.01, and p < 0.001, respectively (dependent on normal distribution ANOVA or Kruskal–Wallis test).

Burn injury increased the expression of factors involved in angiogenesis and remodeling, such as the transcription factor HIF1A and its target genes VEGFA, CXCL12 and FGF2, soon after injury (Fig. 4a–d). Expression levels of CXCL12 and FGF2 remained increased at burn sites until 24 h after burn injury (Fig. 4c,d). LEP expression levels remained unaffected 1 and 4 h after the burn injury but were significantly increased after 24 h (Fig. 4e).

### Burn injury results in an induction of local inflammatory responses

**Figure 5:**
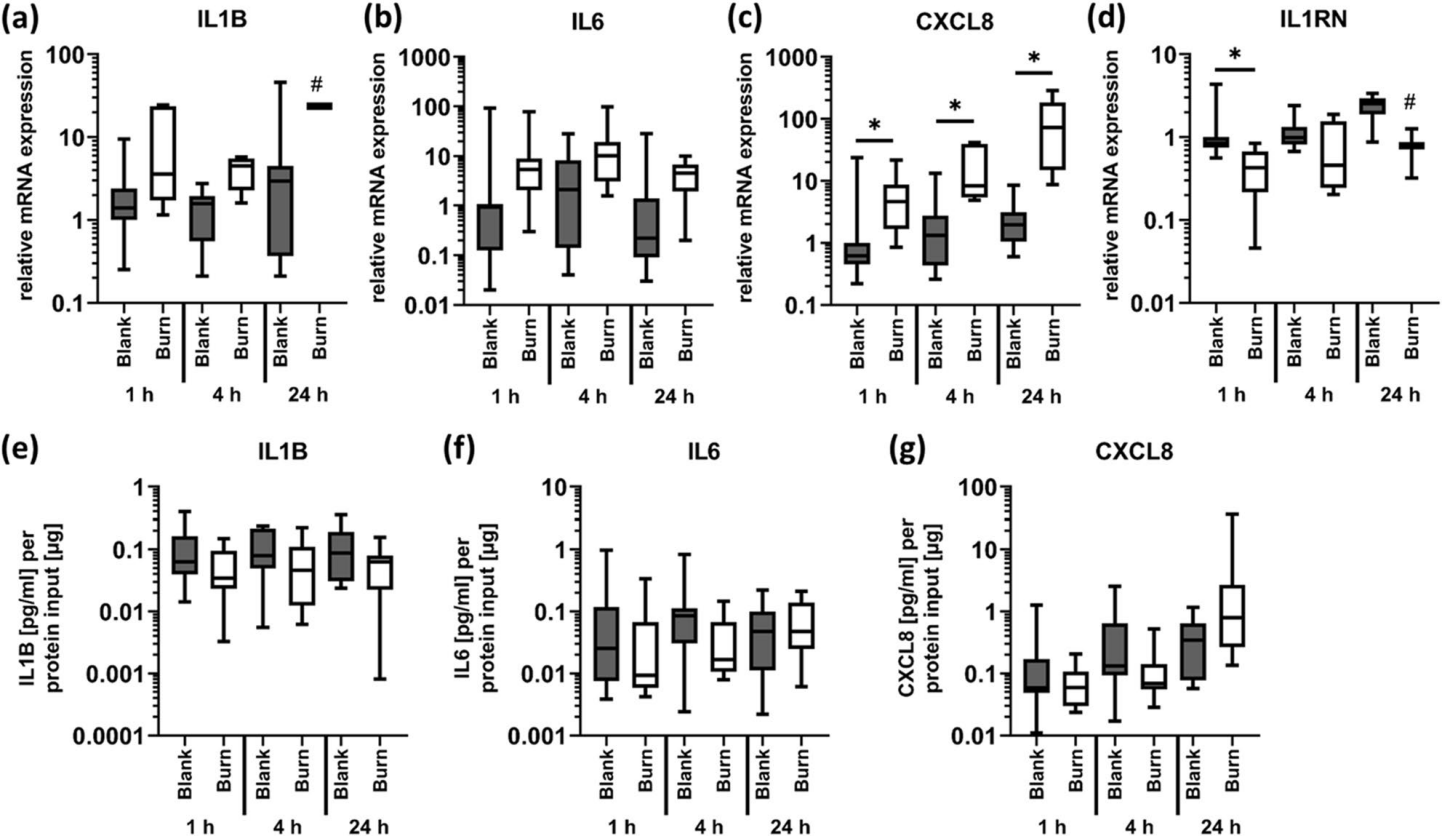
Local inflammatory response after burn injury. Contact burn injuries were created on fresh human skin explants by applying a heated metal block (100 °C). Biopsies were collected after 1, 4 and 24 h. Relative mRNA expression levels of (**a**) IL1B, (**b**) IL6, (**c**) CXCL8 and (**d**) IL1RN were determined by RT-qPCR, normalizing target gene expression to the averaged expression of RPLP0 and TBP. Local cytokine concentrations were measured in homogenized biopsies using the V-PLEX Proinflammatory Panel 1 Human Kit (MSD). Out of 10 candidate analytes, only (**e**) IL1B, (**f**) IL6 and (**g**) CXCL8 could be quantified. Data are presented as median (line) the interquartile range (box) and the minimum and maximum values (whiskers) from three individual skin explants. P-values < 0.05 were considered as statistically significant with *, **, *** indicating p < 0.05, p < 0.01, and p < 0.001, respectively (dependent on normal distribution ANOVA or Kruskal–Wallis test). For specific settings there were less than three values, indicated by #.

Burn injury promoted a local inflammatory response shown by increased expression and protein production of the pro-inflammatory cytokine CXCL8 24 h after injury (Fig. 5c,g). Expression levels of the other pro-inflammatory mediators (IL1B, IL6) did not show significant alterations, but a trend towards an increased expression was observed for IL1B and IL6 mRNA levels (Fig. 5a,b), but not for protein production (Fig. 5e,f). We observed significantly decreased expression levels of IL1RN 1 h after burn injury (Fig. 5d).

### Stimulation with LPS enhances production of pro-inflammatory mediators, but does not alter factors involved in heat shock and tissue regenerative responses

**Figure 6:**
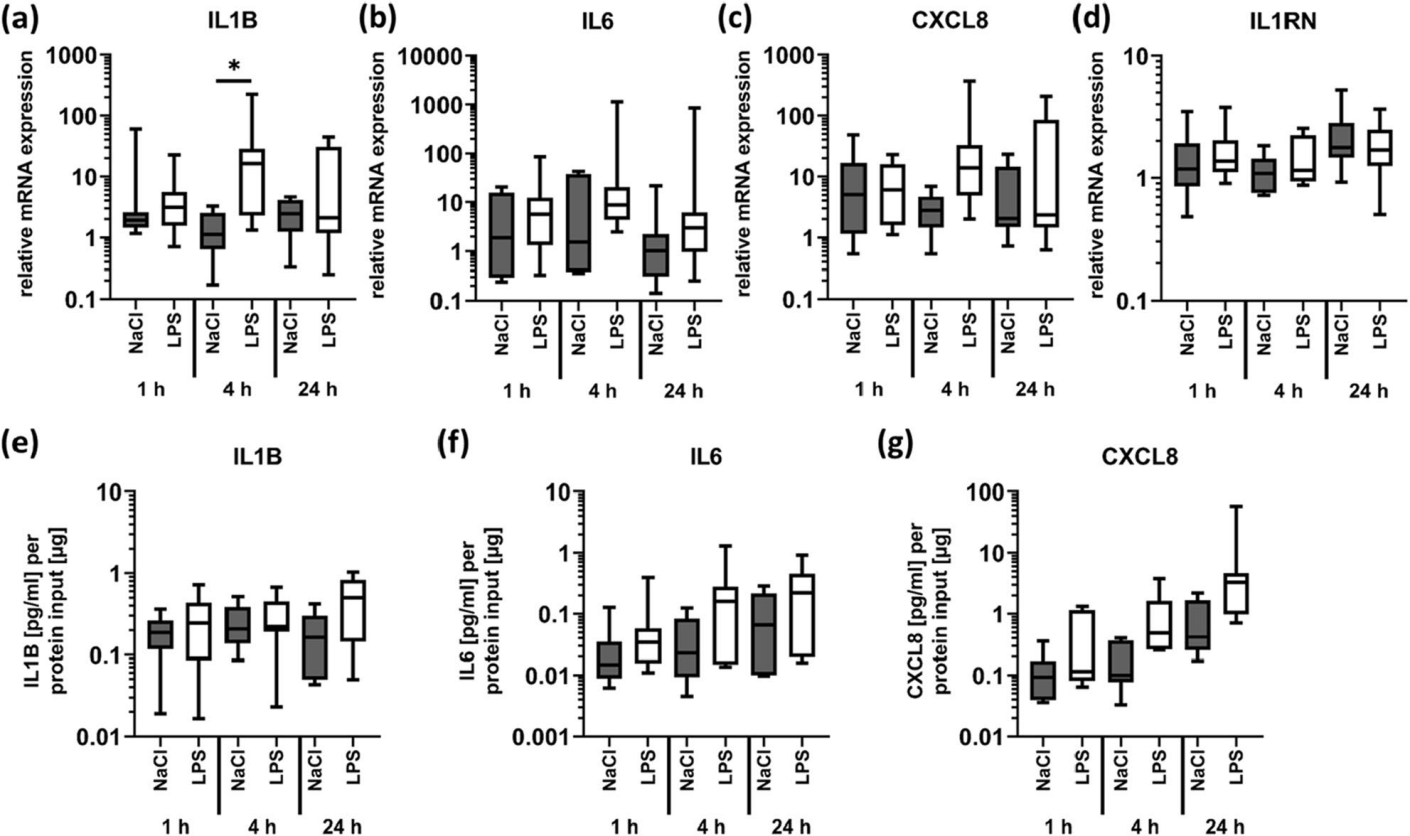
Stimulation with LPS enhances production of pro-inflammatory mediators. LPS (1 µg/ml in 0.9% NaCl; 450 µl/cm^2^) was injected into the dermis of fresh skin explants to analyse inflammatory processes. NaCl was used as a control for the injection trauma. Biopsies were collected after 1, 4 and 24 h. Relative mRNA expression levels of (**a**) IL1B (n = 8), (**b**) IL6 (n = 7), (**c**) CXCL8 (n = 7) and (**d**) IL1RN (n = 8) were determined by RT-qPCR, normalizing target gene expression to the averaged expression of RPLP0 and TBP. Local cytokine concentrations were measured in homogenized biopsies using the V-PLEX Proinflammatory Panel 1 Human Kit (MSD). Out of 10 candidate analytes, only (**e**) IL1B (n = 6), (**f**) IL6 (n = 6) and (**g**) CXCL8 (n = 6) could be quantified. Data are presented as median (line) the interquartile range (box) and the minimum and maximum values (whiskers) from 6–8 individual skin explants. P-values < 0.05 were considered as statistically significant with *, **, *** indicating p < 0.05, p < 0.01, and p < 0.001, respectively (dependent on normal distribution ANOVA or Kruskal–Wallis test).

**Figure 7:**
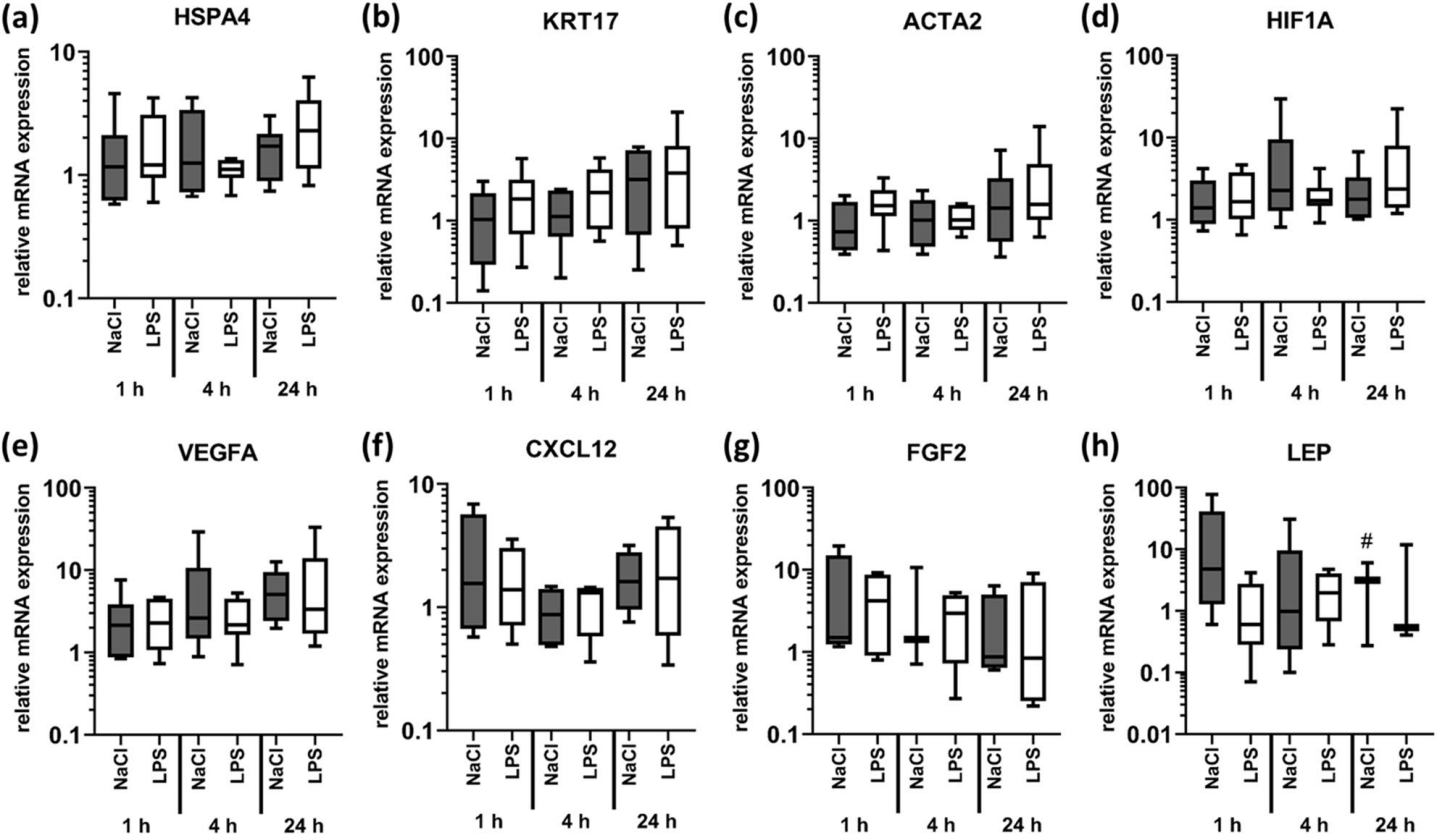
Stimulation with LPS does not alter production of factors involved in heat shock and tissue regeneration responses. LPS (1 µg/ml in 0.9% NaCl; 450 µl/cm^2^) was injected into the dermis of fresh skin explants to analyse inflammatory processes. NaCl was used as a control for the injection trauma. Biopsies were taken after 1, 4 and 24 h. Relative mRNA expression levels of (**a**) HSPA4 (n = 5), (**b**) CXCL12 (n = 4), (**c**) ACTA2 (n = 5), (**d**) KRT17 (n = 5), (**e**) HIF1A (n = 5), (**f**) VEGFA (n = 5), (**g**) FGF2 (n = 4), (**h**) LEP (n = 5) were determined by RT-qPCR, normalizing target gene expression to the averaged expression of RPLP0 and TBP. Data are presented as median (line) the interquartile range (box) and the minimum and maximum values (whiskers) from 4–5 individual skin explants. p-values < 0.05 were considered as statistically significant with *, **, *** indicating p < 0.05, p < 0.01, and p < 0.001, respectively (dependent on normal distribution ANOVA or Kruskal–Wallis test). For a specific setting, less than three values were detected, indicated by #.

The local response to LPS was analyzed in order to investigate an additional inflammatory stimulus independent of tissue disruption. We found a significant up-regulation of the pro-inflammatory cytokine IL1B (Fig. 6a) and a trend for increased expression for IL6 and CXCL8 (Fig. 6b,c) in response to LPS compared with the control site, where a sterile NaCl solution was injected into the dermis. On the protein level, a trend for increased production of CXCL8 was observed 24 h after LPS stimulation compared with the NaCl control site (Fig. 6g), but not for IL1B and IL6 (Fig. 6e,f). No significant differences were observed for IL1RN (Fig. 6d) as well as for the genes involved in heat shock response and regenerative processes (Fig. 7a–h).

## Discussion

In this study, we present a detailed characterization of early processes, including heat shock, inflammatory and regenerative responses, initiated by a partial-thickness burn injury in freshly explanted human skin. Burn injuries are known to trigger a complex cascade of wound healing mechanisms, and with increasing size and depth of the burn injury local responses may result in systemic effects such as SIRS eventually leading to fatal complications^1–4,44^.

Heat shock responses involve the production and activation of heat shock proteins with HSPA4 among the best studied. HSPA4 is found in all cell types and has a chaperone activity as well as signaling functions in innate and adaptive immune responses^45^. Studies in murine models suggested a role for HSPA4 in wound healing after burn or scald injuries^46,47^. However, we found no significant increase in HSPA4 mRNA levels within the first 24 h after burn injury. HSPA4 protein has been described to be increased in the blister fluid of patients with partial thickness burns within the first day after damage but not afterwards^6^. As a chaperone for collagens, SERPINH1 is stably expressed by fibroblasts^7^. Increased amounts of SERPINH1 have been described in response to thermal trauma in rodent models^46,48^. In human studies, SERPINH1 has been used as a marker produced by fibroblasts, where increased expression levels of SERPINH were observed in deep dermal layers compared to superficial dermal layers^49,50^. We did not see any significant alteration in the gene expression pattern of SERPINH1 upon burn injury (data not shown), which may be due to the experimental setup as we did not discriminate between deep and superficial dermal layers, and the observation period was limited to 24 h.

We further analyzed the expression pattern of KRT17, which has been described to be up-regulated in the epidermis during wound healing^51^. We found KRT17 expression to be reduced, which may be attributed to our experimental setting with the burn injury resulting in complete denaturation of the epidermis.

Burn wounds are specifically prone to develop into hypertrophic scars^52^ with increased levels of alpha-smooth muscle actin (ACTA2) in the respective scar tissue of patients or in vivo models^53^. However, we show that the expression of ACTA2 is significantly increased soon after the burn injury in human skin. This increase was shown to be persistent for 24 h after the burn trauma, i.e. for the duration of the experiment. TGFB1 is supposed to be one of the major factors involved in ACTA2 regulation and thus hypertrophic scar development^54,55^. TGFB1 transcription levels were not affected in our experiments. However, TGFB1 is usually released by immune cells and platelets upon injury^56–58^. These TGFB1 sources are unavailable in the skin explants which have no blood circulation. However, TGFB1 is also locally expressed by fibroblasts, with increased levels observed in deep dermal layers^59^. In the skin, TGFB1 is bound as an inactive precursor molecule to the extracellular matrix of the dermis via latency-associated peptide (LAP) and latent TGFB-binding protein (LTBP). Upon tissue disruption, active TGFB1 will be released^56^ activating fibroblasts to differentiate into myofibroblasts producing large amounts of ACTA2^60^.

The transcription factor HIF1A is a key regulator of the cellular response to hypoxia and plays an important role during wound healing as it triggers the expression of angiogenic factors such as VEGFA and CXCL12^61^. We found significant increases in HIF1A expression levels 4 h after burn injury. In line with our data, an early onset of enhanced gene expression of HIF1A was found in a murine wound healing model^8^. HIF1A protein was also detected by immunohistochemistry in the healing marginal zone of burn injuries in mice after 2 days^10^. In the same study, the authors showed enhanced amounts of VEGFA and CXCL12 collocating with HIF1A staining. Using our human ex vivo model, we achieved comparable results, as the expression levels of VEGFA and CXCL12 were also up-regulated after the burn injury which is also true for FGF2—another HIF1A regulated angiogenesis factor^17,18,62^. LEP is an additional factor associated with angiogenesis and wound healing^19–22^, although it is primarily known for its function as a circulating hormone responsible for food intake and energy homeostasis^63,64^. Transcription of LEP was strongly enhanced in the skin 24 h after burn injury in our ex vivo model. Increased levels of LEP were also found in serum samples of burn patients^65,66^. Only recently secreted LEP was detected locally in subcutaneous tissue 3 h after scalding in a rat burn model^67^. At the same time, the authors described reduced LEP mRNA levels in scalded total skin biopsies. LEP expression levels did not decrease in our human ex vivo model at the early measurements 1 and 4 h post injury. However, we observed a strong increase of LEP expression 24 h after a burn injury. To our knowledge, this is the first time to show local LEP mRNA induction in response to a burn injury in a human model and could be investigated as a promising biomarker for severity of burn injuries.

Inflammation is an integral part of wound healing as it is responsible for fending off infections and for clearing the wound area of necrotic cells and debris. Molecules released from necrotic cells, e.g. S100 proteins, high mobility group box 1 (HMGB1), histones, and nucleic acids act as danger-associated molecular patterns (DAMPs) which activate the innate immune system via toll-like receptor signaling^68,69^. The production of inflammatory factors including TNF, IL1B, IL6, CXCL8 and IL1RN is strongly induced at the burn site^12,44,69^. In our ex vivo model, we observed significant induction of the local pro-inflammatory response as mRNA expression of CXCL8 was up-regulated within 1 h after burn injury. A trend for increased mRNA expression of IL1B and IL6 was also found after burn injury. On the protein level, we found no significant changes in response to burns. Importantly, in this ex vivo setting, initial local events were analyzed uncoupled from systemic effects and consequently devoid of infiltrating immune cells. Therefore, the inflammatory responses measured here exclusively reflect the local responses of skin-resident macrophages and dendritic cells, which might explain the extremely low cytokine concentrations observed. In addition, variable regulatory mechanisms, translational efficiencies as well as mRNA and protein half-lives, may account for the poor correlation of mRNA and protein levels^70^. In addition to the pro-inflammatory mediators, we analyzed expression levels of IL1RN which is a natural inhibitor of IL1 signaling. IL1RN has been described to be co-regulated with IL1B and IL1A in response to skin injuries^14^ and increased circulating levels of IL1RN in response to burn injuries have been reported^71,72^. In our experiments, we found a significant local decrease in IL1RN mRNA levels in response to burn injury. Similar to KRT17, IL1RN is locally produced by keratinocytes^73,74^; the observed decrease in IL1RN expression can be explained by the complete denaturation of the epidermis.

In addition to a burn injury, we also analyzed the local responses to bacterial LPS, a TLR-4 ligand, known to activate innate immune system as a cascade of pro- and anti-inflammatory mediators via MyD88-dependent and MyD88-independent pathways^43^. LPS is widely used to mimic bacterial infections allowing for the analysis of inflammatory responses involved in the development of septic conditions^75^. We used LPS to initiate an inflammatory response in the skin to achieve a more subtle stimulus than a partial-thickness burn injury. We found an increase in the expression as well as the production of the pro-inflammatory cytokines IL1B and CXCL8 in response to LPS. Other studies on the inflammatory response of ex vivo skin culture models to LPS have also shown increased levels of various cytokines, e.g. IL1B, IL6 and CXCL8 (IL8), secreted into the medium^32,40^ which corresponds with our data from tissue biopsies. These results suggest that our ex vivo skin model can also be used to investigate early local responses to other stimuli, for example to skin infections.

### Limitations

Our ex vivo skin model has a great potential for the analysis of different skin pathologies, as the model allows for physiological local responses and reflects the complex interactions between the epidermal and dermal cells in a fully human setting. However, this ex vivo approach is limited by the lack of a blood circulation in the explanted skin. Burn-induced erythema and edema formation cannot be observed (Fig. 1a). This has to be considered when planning the experimental setup, as these are important factors for burn wound progression. As mentioned above, the inflammatory responses analyzed in this ex vivo setting exclusively reflect the local responses of skin-resident immune cells, as platelets, neutrophils and monocytes cannot infiltrate the site of burn injury.

The used ex vivo model has to be thoroughly standardized since we found considerable variations between individual donors^40^. To obtain significant results, the number of experiments has to be planned accordingly. In our setting, we used eight skin explants for LPS injection (donors 1–8), and three explants for burn injuries (donors 5–7). Burns are a very strong stimulus activating numerous pathways of inflammation and tissue regeneration^44^. Burn injuries resulted in a significant inflammatory and tissue damage response. In contrast, LPS acted as a more subtle stimulus after injection of a moderate concentration (1 µg/ml) and subsequently higher inter-individual variations required a larger number of explants for the LPS experiment.

Another limitation, which is also intrinsic to the use of human skin explants, is the short experimental observation period of approximately 36 h. We did not observe any major degradation-mediated morphological changes in healthy skin areas in the histological analysis (Fig. 2a). In addition, ultrastructural analysis indicated that the skin integrity is preserved over the entire duration of the experiment. However, we found small but significant changes in some of the molecular markers after 24 h in untreated skin samples. For example, the stress marker HSPA4 was slightly upregulated, as well as the regenerative maturation marker KRT17^32^, whereas expression of SERPINH1 decreased slightly over time (supplementary figure S1). Also for HIF1A and VEGFA (but not for FGF2, CXCL12 or LEP) as well as for CXCL8 and IL1RN (but not for IL1B and IL6) significantly increased mRNA levels were observed. Expression levels of TGFB1 and ACTA2 were not altered in blank skin over time. Since especially the epidermis relies more on atmospheric oxygen rather than on oxygenation via vasculature and since the skin is always under mild hypoxic conditions^76^, oxygen deprivation for the duration of the experiment probably did not interfere with the outcome of the experiment. The ischemic conditions most likely resulted in glucose deprivation. HIF1A has also been described to be involved in glucose sensing and regulation of glucose metabolism at least in immune cells and cancer^77^. Thus, we speculate that the increase in HIF1A expression over time reflects the lack of nutrients, and, to a minor extent, some lack of oxygen. Throughout the experiment, dehydration of the skin was avoided by the constantly moist conditions in the climate chamber. Therefore, we limited the duration of the ex vivo experiments to a total of 36 h.

Our results describe local inflammatory and regenerative responses to burns in skin that was derived only from the abdominal region. Skin characteristics, including the thickness and elasticity of the skin, or the amount and composition of the subcutaneous adipose tissue vary greatly with anatomical topology^78^. However, the basic responses to tissue disruption or inflammation, as analyzed in this study, are highly conserved^68^. Although no skin explants from other anatomical regions were used in our study, we presume that early local reactions would be similar in skin derived from other sites, e.g. the face, chest or fingers. Moreover, only skin explants from female donors were available for our study, as the majority of patients undergoing this kind of elective surgeries are female. Sex-based differences in burn outcome is a matter of debate, as some studies reported increased mortality in women, whereas others did not find any differences^79–83^.

Despite these limitations, this ex vivo skin model offers at least two major advantages over conventional in vivo and in vitro models. First, inter-species variations in skin structure and immune responses are avoided, since skin explants from human donors are used. Second, an intact 3D skin structure is maintained including the complete resident immune system, a feature that is currently not available in vitro^84^.

The ex vivo model presented here provides a valuable tool to analyze early steps in the healing processes of burn injuries and other pathological skin conditions such as inflammatory skin diseases. Since the complex pathological processes accompanying burn injuries ultimately stem from local tissue damage responses, it is important to characterize the profile of local inflammation and tissue regeneration markers. Further studies by using this ex vivo model will provide a more detailed insight into the local processes induced by burns paving the way for a better predictability of burn injury outcomes and eventually contributing to a decreased burn-related mortality. It is well established that severe burn injury is able to initiate SIRS which can result in organ failure and premature death^25,85^. Still, underlying mechanisms of SIRS development are not yet fully understood. Analyses of local events in response to burns are underrepresented in the literature, but could provide valuable insights^86^. We think that local inflammatory responses after burn injury might be critically involved in starting a severe systemic inflammatory response cascade. Although animal models provide invaluable insight into pathways influenced by burn injuries, differences in skin structure and immune response do not completely mimic the human situation^32^. Therefore, the ex vivo model proposed here could help to elucidate early, strictly local responses to burn injuries. The data obtained could be correlated to systemic data collected from burn patients with the future aim to establish new markers for early SIRS detection.

## Methods

### Tissue samples

Adult human skin explants were obtained from eight healthy female donors aged between 30 and 64 (mean age: 42.5 ± 12) years undergoing abdominoplasty or circumferential body lift surgery at the Division of Plastic, Aesthetic and Reconstructive Surgery, Department of Surgery, Medical University of Graz, Austria. All methods were carried out in accordance with Declaration of Helsinki principles. The study was approved by the Ethical Committee of the Medical University of Graz (project identification: EK: 28-151 ex 15/16; approval extended until 22.12.2020). All subjects provided written informed consent; the Biobank Graz (Medical University of Graz, Austria) was responsible for tissue transport and pseudonymization.

### Human ex vivo skin model of burn injury and inflammation

Fresh skin explants were immobilized, cleaned and transferred into a climate chamber, which provided standardized environmental conditions (32 ± 2 °C; 40–60% humidity). After 1 h of acclimatization, treatment sites on the skin were marked using a template to standardize the stimulation pattern (Fig. 1). Contact burn injuries were created by application of heated metal blocks (Zultner Metall GmbH, Graz, Austria; 5 × 5 cm; 1.9 kg; 100 °C) onto the skin for ten seconds without any additional pressure. In order to analyze inflammatory processes independent of tissue disruption, lipopolysaccharide (LPS; *E. coli* O5:B55, Invivogen, Toulouse, France; 1 µg/ml in 0.9% NaCl, Fresenius Kabi, Bad Homburg, Germany) a well-known TLR4-ligand^43^ was injected into the dermis. In order to control for the injection trauma, 0.9% NaCl was used; 450 µl of LPS or NaCl solution were injected per 1 cm^2^ in 9 single points of 50 µl each. The LPS experiment was performed eight times (donors 1–8), the burn experiment three times (donors 5–7). For RNA and protein analyses, as well as for histology punch biopsies (8 mm) were taken at 1, 4 and 24 h after burn injury, injection or blank skin sites. Biopsies were either fixed for histology, TEM or snap frozen on dry ice and stored at − 80 °C until further processing.

### Histology

Skin biopsies were fixed in 10% neutrally buffered formalin solution and were processed in a Tissue-Tek VIP (Sakura, CA, USA). Three µm thick sections were prepared and attached to charged glass slides (Menzel Superfrost Plus, Thermo Fisher Scientific, MA, USA) and stained with haematoxylin and eosin. Images were captured with an Aperio ScanScope AT digital slide scanner (Leica Biosystems, Wetzlar, Germany) at 40-fold magnification.

### Transmission electron microscopy

Minutien pins were used to immobilize skin biopsies on gelatin coated plates. Samples were transferred to 2.5% (wt/vol) glutaraldehyde and 2% (wt/vol) paraformaldehyde in 0.1 M phosphate buffer, pH 7.4, for 24 h, then were postfixed in 2% (wt/vol) osmium tetroxide at room temperature for 3 h. After washing for 2 h in 0.1 M phosphate buffer the specimens were dehydrated in a graded series of ethanol (50%, 70%, 80%, 96%, 100% p.a.). Samples were infiltrated with propylene oxide/TAAB (Agar Scientific, Essex, UK) embedding resin (propylene oxide 1 h room temperature, propylene oxide/TAAB 1:1 3 h room temperature, propylene oxide/TAAB 1:3 overnight at 4 °C) and finally embedded in pure resin (2 × 1.5 h 48 °C, polymerized 48 h at 60 °C).

Ultrathin Sects. (70 nm thick) were cut with a UC 7 Ultramicrotome (Leica Microsystems, Wetzlar, Germany) and stained with lead citrate for 5 and platinum blue for 15 min. Serial EM pictures were taken using a Tecnai G2 20 transmission electron microscope (Field Electron and Ion Company, OR, USA) with a Gatan ultrascan 1000 charge coupled device (CCD) camera (temperature − 20 °C; acquisition software Digital Micrograph; Gatan, CA, USA). Acceleration voltage was 120 kV.

### Gene expression analysis

Skin biopsies were homogenized in Qiazol using MagNa Lyser Beads and the MagNA Lyser instrument (Roche, Basel, Switzerland). Immediately after homogenization, RNA was isolated using RNAeasy Lipid Tissue Mini Kit (Qiagen, Hilden, Germany) according to the instructions provided by the manufacturer. RNA concentration was determined on a NanoDrop microvolume spectrophotometer (Thermo Fisher Scientific, MA, USA). For cDNA synthesis (iScript gDNA clear kit, Biorad, CA, USA) 0.5–1 μg of total RNA was used.

Predesigned TaqMan assays for the genes of interest and the endogenous control genes (Table 1) as well as the TaqMan Gene Expression Master Mix were purchased from Thermo Fisher Scientific, MA, USA; all qPCR experiments were run on a CFX384 cycler (Bio-Rad, CA, USA) using standard conditions according to the manufacturer’s instructions. Relative gene expression was calculated using the delta-delta-Cq method^87^, normalizing target gene expression to the averaged Cq of the two endogenous control genes (RPLP0, TBP) and calibrating all samples to a blank skin sample taken 1 h after stimulation. N-fold expression levels are presented as median (line) the interquartile range (box) and the minimum and maximum values (whiskers) from at least three independent experiments. Numbers of individual skin explants (n) are indicated for specific results in the respective figure legends. Due to limited sample amounts, not all genes were analyzed for all experiments. Samples were excluded from further analysis when at least one of the two endogenous control genes failed to give a Cq value.

**Table 1:** Taqman gene expression assays (Thermo Fisher Scientific, MA, USA).

Gene	Name; commonly used aliases	Cat. No.
IL6	Interleukin 6	Hs00174131_m1
CXCL8	C–X–C motif chemokine ligand 8; interleukin 8 (IL8)	Hs00174103_m1
TNF	Tumor necrosis factor; TNF alpha (TNFa)	Hs00174128_m1
IL1B	Interleukin 1 beta	Hs01555410_m1
IL1RN	Interleukin 1 receptor antagonist	Hs00893626_m1
TGFB1	Transforming growth factor beta 1	Hs00998133_m1
FGF2	Fibroblast growth factor 2; basic FGF (bFGF)	Hs00266645_m1
VEGFA	Vascular endothelial growth factor A	Hs00900055_m1
ACTA2	Actin alpha 2, smooth muscle; alpha smooth muscle actin (aSMA)	Hs00426835_g1
KRT17	Keratin 17	Hs00356958_m1
HSPA4	Heat shock protein family A (Hsp70) member 4; HSP70	Hs00382884_m1
SERPINH1	Serpin family H member 1; HSP47	Hs01060397_g1
HIF1a	Hypoxia inducible factor 1 subunit alpha	Hs00153153_m1
CXCL12	C–X–C motif chemokine ligand 12; stromal cell-derived factor 1 (SDF1)	Hs03676656_mH
LEP	Leptin	Hs00174877_m1
RPLP0	Ribosomal protein lateral stalk subunit P0	Hs00420895_gH
TBP	TATA-box binding protein	Hs00427620_m1

### Cytokine analysis

Human skin biopsies were homogenized in 500–1000 µl buffer (50 mM Tris HCl pH 7.6 supplemented with 1:100 Protease Inhibitor Single Use Cocktail and 1:100 Phosphatase Inhibitor Cocktail; both Thermo Fisher Scientific, MA, USA) using the Bead Ruptor Elite device (Omni International, GA, USA) with two 30 s cycles (7.3 m/s). Samples were incubated on ice for 2 min between cycles and prior to centrifugation (10 min, 10,000 *g*, 4 °C). Homogenates were aliquoted and stored at − 80 °C. Total protein concentration was determined using a bicinchoninic acid assay (Pierce BCA Protein Assay Kit, Thermo Fisher Scientific MA, USA). Briefly, 25 µl of homogenized sample were mixed with working reagent in a microplate for 30 s. The plate was incubated at 37 °C for 30 min and after cooling to room temperature, the absorbance was measured at 570 nm, protein concentrations were determined in comparison to a bovine serum albumin (BSA) derived standard curve.

The V-PLEX Pro-inflammatory Panel 1 Human Kit (Meso Scale Diagnostics, MSD, MD, USA) was used to determine the concentrations of 10 cytokines in parallel (IFNG, IL1B, IL2, IL4, IL6, IL8, IL10, IL12p70, IL13, TNF) in the skin homogenates according to the manufacturer’s instructions. The plates were read on the MESO QuickPlexTM SQ 120 instrument (MSD, MD, USA). Cytokine concentrations (pg/ml) were normalized to protein input (µg). Results are presented as median (line) the interquartile range (box) and the minimum and maximum values (whiskers) from at least three independent experiments. Numbers of individual skin explants (n) are indicated for specific results in the respective figure legends. Due to limited sample amounts, cytokine production was analyzed for all experiments.

### Statistical analysis

For the statistical analysis of qPCR and MSD data, GraphPad Prism 8.4.2 software for Windows (GraphPad Software, CA, USA) was used. Data were tested for log-normal distribution (Shapiro–Wilk); data that were found to be log-normally distributed were tested with a one-way-ANOVA (Sidak’s test for multiple comparisons) for significant differences. Data that did not show log-normal distribution were tested for significant differences between groups using the non-parametric Kruskal–Wallis test, with Dunn’s test for multiple comparisons; p-values < 0.05 were considered as statistically significant with *, **, *** indicating p < 0.05, p < 0.01, and p < 0.001 respectively.

## Supplementary Information

Supplementary Figure S1.

## Data Availability

The datasets generated and analysed during the current study are available from the corresponding author on reasonable request. All data generated and analysed during this study are included in this published article.
