# Supplementary material for: A novel human ex vivo skin model to study early local responses to burn injuries
Source: Sci Rep. 2021 Jan 11;11:364. doi: 10.1038/s41598-020-79683-3 (PMC7801530; doi:10.1038/s41598-020-79683-3)
Supplement: Supplementary file 1 — Supplementary Figure S1. [file 41598_2020_79683_MOESM1_ESM.docx]

Supplementary Data:

**A Novel Human Ex Vivo Skin Model to Study Early Local Responses to Burn Injuries**

# Elisabeth Hofmann ^1,2^, Julia Fink ^1^, Anita Eberl ^3^, Eva-Maria Prugger ^3^, Dagmar Kolb ^4,5^, Hanna Luze ^1,2^, Simon Schwingenschuh ^3^, Thomas Birngruber ^3^, Christoph Magnes ^3^, Selma I. Mautner ^2,3,6^, Lars-Peter Kamolz ^1,2^, and Petra Kotzbeck ^1,2,6^*


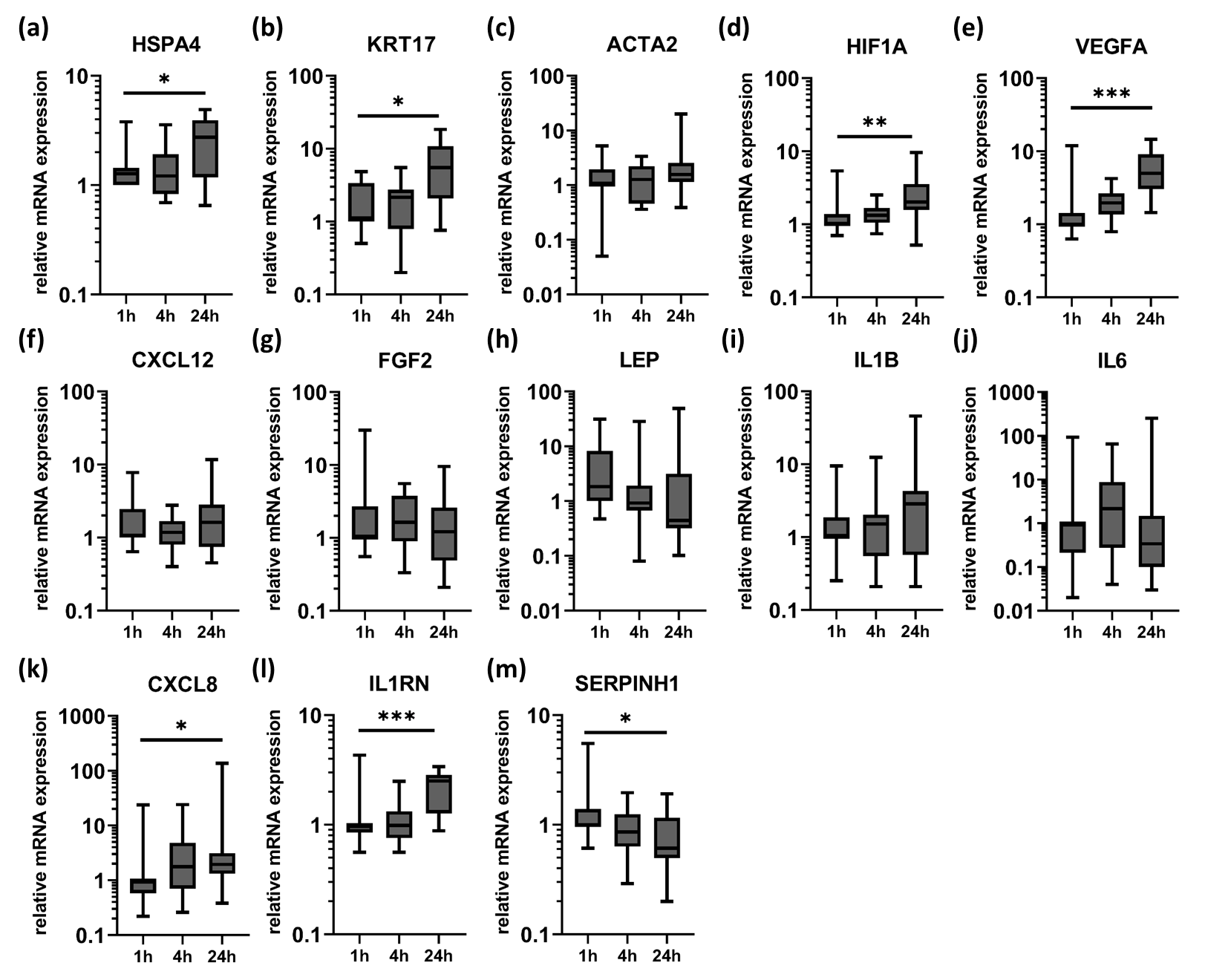


**Supplementary Figure S1**. **Molecular changes in blank skin samples over 24 hours.** Skin explants from plastic surgeries were used for a burn or inflammation model. Biopsies were collected from unburnt, blank skin after 1, 4 and 24 hours, respectively. Relative mRNA expression levels of (a) HSPA4, (b) KRT17, (c) ACTA2, (d) HIF1A, (e) VEGFA, (f) CXCL12, (g) FGF2, (h) LEP, (i) IL1B, (j) IL6, (k) CXCL8, (l) IL1RN and (m) SERPINH1 were determined by RT-qPCR, normalizing target gene expression to the averaged expression of RPLP0 and TBP. Data are presented as median (line) the interquartile range (box) and the minimum and maximum values (whiskers). P-values < 0.05 were considered as statistically significant with *, **, *** indicating p < 0.05, p < 0.01, and p < 0,001, respectively (dependent on normal distribution ANOVA or Kruskal-Wallis test).
